# HARNESSING THE POWER OF OLDER AMERICANS: INDEPENDENCE CENTER NETWORKS TO DEVELOP TECHNOLOGIES PROMOTING MOBILITY

**DOI:** 10.1093/geroni/igz038.029

**Published:** 2019-11-08

**Authors:** Katherine S Hall, Kevin Caves, Neil Alexander

**Affiliations:** 1 Durham Veterans Affairs Health Care System, Durham, North Carolina, United States; 2 Duke University, Durham, United States; 3 University of Michigan, Ann Arbor, Michigan, United States

## Abstract

Information communication technology (ICT) refers to various technologies encompassing software, networking, the Internet, telecommunications, information systems, and more. As healthcare organizations adopt ICT devices and platforms, patients and providers will have more tools available to improve access to monitoring, telehealth, and timely interventions. The use of alternative methods of collecting, recording, and displaying data (e.g., smart speakers, chat bots, wearables) promise to improve health outcomes for the older adult population. As the U.S. population ages, opportunities for development designed specifically for older adults should be a focus for healthcare organizations. While there are challenges and barriers to enabling new technology within this population, research shows that older adults are adopting new technology. This symposium is focused on these emerging technologies and will showcase diverse examples of ICT implemented across various older adult populations and clinical application areas. The first paper describes the validation of Gaitbox, a walking speed measurement device. The second paper describes using multiple sensors to capture real-world loss of balance and recovery responses. The third paper reports the feasibility of using fitness gamification with a Virtual Reality Treadmill in older adults. The fourth paper describes a smartphone-based assessment of dual task standing and walking. The fifth paper describes wearable sensor-based assessment of falls risk of Timed Up-and-Go test. The Claude D. Pepper Centers maintain year-round coordination and collaboration through a national coordinating center. This powerful network, working towards the common goal of improving the lives of older Americans, has sparked technologic advances that will be highlighted here.

